# Association of CCL2, CCR5, ELMO1, and IL8 Polymorphism with Diabetic Nephropathy in Malaysian Type 2 Diabetic Patients

**DOI:** 10.1155/2019/2053015

**Published:** 2019-01-01

**Authors:** Mohd Jokha Yahya, Patimah binti Ismail, Norshariza binti Nordin, Abdah binti Md Akim, Wan Shaariah binti Md. Yusuf, Noor Lita binti Adam, Maryam Jamielah Yusoff

**Affiliations:** ^1^Department of Biomedical Sciences, Faculty of Medicine and Health Sciences, Universiti Putra Malaysia, Malaysia; ^2^Department of Human Development and Growth, Faculty of Medicine and Health Sciences, Universiti Putra Malaysia, Malaysia; ^3^Department of Medicine (Endocrinology & Nephrology), Hospital Tuanku Ja'afar, Malaysia

## Abstract

The unique variants or biomarkers of individuals help to understand the pathogenesis as well as the potential risk of individuals or patients to diabetic nephropathy (DN). The aim of this study was to investigate the association of a genetic polymorphism of monocyte chemoattractant protein-1 (CCL2-rs3917887), chemokine receptor 5 (CCR5-rs1799987), engulfment and cell mortality (ELMO1-rs74130), and interleukin-8 (IL8-rs4073) with the development of DN among Malaysian type 2 diabetes mellitus (T2DM) patients. More than one thousand diabetic patients were examined and a total of 652 T2DM patients were tested comprising 227 Malays (nonnephrotic=96 and nephrotic=131), 203 Chinese (nonnephrotic=95 and nephrotic=108), and 222 Indians (nonnephrotic=136 and nephrotic=86). DNA Sequenom mass ARRAY was employed to identify polymorphisms in CCL2, CCR5, ELMO1, and IL8 genes. DNA was extracted from the secondary blood samples taken from the T2DM patients. The alleles and genotypes were tested using four genetic models and the best mode of inheritance was chosen. CCR5 rs1799987 (G>A) showed strong association with the development of diabetic nephropathy only among the Chinese with OR=6.71 (2.55-17.68) 95% CI while IL8 rs4073 (T>A) showed association with nephropathy only among the Indians with OR=1.57 (0.66-3.71) 95% CI. The additive model was the best model for the mode of inheritance of all the genes. The contribution of genetic variants differs across ethnic groups or background. Further studies which involve environmental risk factors should be taken into consideration.

## 1. Introduction

The most common modifiable risk factors for most chronic diseases include, but not limited to, dyslipidemia, hypertension, and glycemic control while factors such as age, race, and genetic profile are generally unmodifiable [1–3].

Diabetes nephropathy (DN) has become a major determinant of morbidity and mortality in diabetic patients worldwide in addition to being the most common cause of end-stage renal disease (ESRD) [4]. Though the main causes of DN are hemodynamic and metabolic factors, it has recently been suggested that DN is an inflammatory process involving immune cells [5]. For instance, hyperglycemia, free fatty acids (FFA), and obesity may activate nuclear factor *κ*B (NF-*κ*B) through protein kinase C (PKC) and reactive oxygen species (ROS) to rapidly stimulate the expression of cytokines which stimulate some genes that promote the development of DN [6]. Thus, the pathogenesis of DN via increased vascular inflammation and fibrosis has implicated inflammatory cells, cytokines, and profibrotic growth factors such as transforming growth factor-*β* (TGF-*β*), monocyte chemoattractant protein-1 (MCP-1), interleukin-1 (IL-1), interleukin-6 (IL-6), and interleukin-18 (IL-18), among others [7]. The activation of PKC, oxidative stress, and advanced glycation end products (AGEs) due to the diabetic state also increases the production of cytokines such as TNF-*α* and interleukins, which stimulate the production of MCP-1, also known as CCL2. PKC also directly induces the production of NF-țB which promotes the production of proinflammatory proteins and extracellular matrix turnover such as thrombospondin 1, chemokine CCL2, osteopontin, fibronectin, decorin, plasminogen activator inhibitor 1, and aldose reductase [8].

CCR5 is a *β*-chemokine receptor expressed on the surface of the monocytes for its ligands or known as regulated on activation of normal T cell expressed and secreted (RANTES) on mesangial cells. It is normally involved in the migration of monocytes, NK cells, and some T-cells to the inflammation site [9, 10]. An SNP of CCR5 (G59029A) gene is also registered as rs1799987. Its gene is located on the short arm of chromosome 3 (3p21.31) [11] at the promoter region. Though there are conflicting and inconclusive reports on the association of CCR5 gene promoter polymorphism with the risk of DN, the polymorphism in CCR5 might affect individual susceptibility to DN [12]. The gene, however, has been reported to have an association to nephropathy in T2DM among the Japanese and Asian Indian population [13–16]. Some polymorphisms of CCR5 gene have been reported to affect the severity of multiple autoimmune and infectious diseases through mediating inflammatory responses [17]. According to Nazir et al. [10], CCR5 rs1799987 is the most studied genetic variant in inflammatory cytokines with the genetic variant rs1799987 in CCR5 gene A allele being the risk factor for diabetic nephropathy. The G allele of rs1799987, however, is considered as a protective allele [18]. CCL2 is also recognized as monocyte chemoattractant protein-1 (MCP-1) is the strongest known chemotactic factor for monocytes and is upregulated in DN. The CCL2 gene is located on chromosome 17q11.1–q21.1. The insertion-deletion sequence located in intron 1 (AGCTCTCCTTCTC/-) is registered as rs3917887 and was found to be significantly correlated to TDM2 among the North and South Indian population [13]. The blockade of CCL2/CCR2 signaling by RS102895 reportedly ameliorates diabetic nephropathy by improving blood glucose levels and preventing CCL2/CCR2 signaling from altering renal nephrin and VEGF expressions through blocking macrophage infiltration, inflammation, and oxidative stress in type 2 diabetic mice [19].

Interleukin-8 is a potent chemokine inducing chemotaxis which recruits and activates acute inflammatory cells such as neutrophils, basophils, and T lymphocytes [20, 21]. The IL8 urinary level was found to be increased in T2DM patients with nephropathy in stage 1 and stage 2 [22]. The high level of IL-8 is due to the IL8 T-251A polymorphism (rs4073) on chromosome 4q12–13 that lies at the regulatory region also known as a promoter that affects the gene expression [23]. Ahluwalia et al. [13] have reported an association between nephropathy and IL-8 gene polymorphism in North and South Indians.

Engulfment And Cell Motility 1 (ELMO1) is a protein consisting of 720 amino acids. It is encoded by the ELMO1 gene on chromosome 7p14 with 22 exons. ELMO1 encodes for one of the ELMO proteins which promotes phagocytosis and cell migration by interacting with the cytokinesis protein. Polymorphisms in ELMO1 are strongly associated with susceptibility to DN [24, 25]. ELMO1 variants have been studied; the most strongly associated, however, is rs741301 among the Chinese population [26], African Americans [27], and Japanese [28]. 

In general, the roles of the aforementioned four proinflammatory genes in the development of DN have been established in some races. Therefore, this study is aimed at investigating the association of a genetic polymorphism of CCL2-rs3917887, CCR5-rs1799987, ELMO1-rs74130, and IL8-rs4073 with the development of DN among Malaysian type 2 diabetes mellitus (T2DM) patients.

## 2. Materials and Methods

This study employed a case-control design using cases with nephropathy and without nephropathy control groups of T2DM patients. The study was approved by the National Medical Research Register (NMRR) of the Ministry of Health Malaysia (KKM) with the reference number: (2) DLM.KKM/NIHSEC/08/0804/P12-519). The study also conforms to the items of the Declaration of Helsinki. All the subjects gave a written and signed informed consent. The subjects were recruited from the outpatients of the Medical Clinic of Hospital Tuanku Ja'afar Seremban (HTJS), Negeri Sembilan, Malaysia. The same patient cohort participated in another study for oxidative stress-related polymorphisms by Yahya et al. [29].

### 2.1. Sampling Method

From over 1000 T2DM patients' records screened from the medical outpatient department in HTJS, only 820 subjects were found to be suitable for the research with only 652 eventually selected with 203, 227, and 222 Chinese, Malays, and Indians, respectively. The selection of the subjects was based on the inclusion criteria for nephropathy (case) and nonnephropathy (control) following expert opinions of the specialists in the endocrinology and nephrology clinics. Only interested patients with signed consent were allowed to participate in this research. The exclusion criteria for both the case and control include biologically related patients, those with age onset ≤ 35 years, diabetes duration ≤ 10 years, normal fasting glucose level, nondiabetic and normal albumin excretion rate, patients without renal symptoms with a duration of < 10 years of diabetes, unclear cause of renal damage, and ESRD or Non-ESRD of T1DM patients as well as patients with glycated hemoglobin (HbA1c) <6.5%.

### 2.2. DNA Extraction and Genotyping

The samples used were secondary blood samples of T2DM patients who routinely visit the clinic. The samples were taken with ethylenediaminetetraacetic acid (EDTA) anticoagulant vacutainer tubes and stored at −20°C for further extraction and analysis. Both the patients' demography and most recent biochemical results were obtained from the patients' records and laboratory information system (LIS) in the Department of Pathology, respectively. Commercial DNA extraction kit (QIAGEN, USA) was used for the genomic DNA extraction while Sequenom Mass ARRAY iPLEX platform was employed for the SNPs genotyping. The derivation of the primer was done based on the work of Ahluwalia et al. (2011).  Table 1 shows the sequence of primers and size of the PCR products used for the genotyping.

### 2.3. Statistical Analysis

The Statistical Package for the Social Sciences version 17.0 (SPSS17.0) was used for all the statistical genetic analyses. Descriptive and inferential statistics were used to compare between the cases and controls for the frequencies of alleles and genotypes. One-way analysis of variance (ANOVA) was also used to test the differences between the clinical data. The conformation of the controls to Hardy-Weinberg Equilibrium (HWE) and the variants distributions were tested using Pearson's Chi-square goodness-of-fit test. Conventional Pearson's Chi-square test for independence with 2df was used for the genotype frequencies for each SNP to determine their associations with T2DM nephropathy. The statistical significance was set at p<0.05 (two-tailed). The Fisher Exact tests 2 × 3 and 2 × 2 were performed only when more than 20% of the cells had expected values less than 5 and when more than 20% of the cells had expected values less than 10, respectively. Three types of the model using Chi-square with 1 df as well as Cochran-Armitage trend test were also used to determine the significant SNPs. Odds ratio (OR) with corresponding 95% confidence interval (CI) was employed for the strength of association or the risk of developing diabetic nephropathy. VassarStats was used to calculate the OR. And the model with the least p-value was chosen to determine the mode of inheritance.

## 3. Results

### 3.1. Characteristics of T2DM Sampling Subjects

The Malays, Chinese, and Indians in this study were 227, 203, and 222, respectively, making a total of 652 T2DM patients. As reported earlier by Yahya et al. [29], the age ranges of the Malay, Chinese and Indian T2DM subjects were 32–83, 36–89, and 35–86 years old, respectively, while the mean ages were 59.0±8.23, 63.28±11.56, and 61.33±10.1, respectively.

In line with the aim of this study to only observe the effects of polymorphism as risk factors, the development of nephropathy was, therefore, not staged among the T2DM nephropathy patients and the basis of selection was mainly with the AER more than 300 mg/24hr. In the clinical demographics of the subjects in this study (data not shown), all the races showed similar characteristics where the significant difference between the case and control was observed in AER, total cholesterol, and HDL (p<0.05). The biochemical observation among the races is shown in Table 2. There was no difference in duration of diabetes, glycated hemoglobin, fasting blood glucose, LDL, and triglycerides level (p>0.05) in the T2DM subjects case and control.

The statistical analysis by one-way ANOVA did not indicate a significant difference in the chemistry results (p>0.05) except for the glycated hemoglobin (HbA1c) concentration (p<0.01). The Chinese had the worst glycemic control in both case and control. There is a significant difference in glycemic control value among the races. The sampling was not randomly chosen; therefore the results might not reflect the true population clinical status among the races. Samples were selectively chosen to fulfill criteria of the research interest and might cause bias results.

### 3.2. Genotyping of Polymorphisms

All the polymorphisms were done by the mass array, and the homozygous and heterozygous genotype were interpreted by the observation of peaks produced in the chromatogram. The results are shown in Tables 3, 4, and 5.

### 3.3. Chi-Square Test of Genotype and Allele Association

In this study, all controls were tested for HWE and the results showed that all the controls were in the HWE with p>0.05 as shown in Table 6. Pearson's Chi-square test for independence was done to determine the association between variants and nephropathy (Table 7). The outcome of the Chi-square at df=2 with p<0.05 was accepted as a statistically significant association (a difference in genotype frequency between the case and control) of nephropathy with the gene polymorphism of the genotype and allele. Fisher Exact test probability was tested when more than 20% of the cells had the expected value < 5.

### 3.4. Dominant and Recessive Model

The association of the above genotypes was then stratified against the dominant and recessive model as shown in Table 7. Only the rs1799987 showed significant association in both dominant (p=9.2x10^−5^) and recessive (p=0.0021) models among the Chinese only.

### 3.5. Cochran-Armitage Trend Test

For best understanding of the mode of inheritance and to calculate the additive model association, the association was tested for trend using Cochran-Armitage trend test (C-ATT). From Table 8, the best model fit for all the variants was the additive. The best model was chosen based on the least p-value compared to the other models.

The OR of the associated polymorphisms is skewed towards increasing risk of nephropathy for T2DM patients among the Malaysian. The exposure to the tested polymorphisms is not absolute as there are many other related polymorphisms that need to be incorporated.

From Table 9, individuals with homozygous SNP of rs1799987, CCR5, have the strongest association to nephropathy in T2DM (p=1.0x10^−5^) with OR=6.71, 95% CI 2.55-17.68) but this is only among the Chinese. The weakest association would be the rs4073, IL8 (p= with OR=1.57, 95% CI 0.66-3.71) that is only applicable among the Indians, odd ratio (OR), 95% CI.

## 4. Discussion

### 4.1. Genetic Association and Correlation

In this study, four polymorphisms of four different genes were tested to confirm variants that might be the risk factors to increase the susceptibility to DN among Malaysians. Among the four proinflammatory genes studied, only the CCR5 and IL-8 were significant among the Chinese and Indians, respectively.

In the present study, CCR5 59029 A allele has a significant and strong association with nephropathy in T2DM Malaysian Chinese only. Evidently, 50% of A allele carriers were found in the case group while only 28.9% of the carriers were found in the control group. The CCR5 59029A-genotype has been shown to be associated with increased CCR5 expression. According to the literature, CCR5 59029A increased CCR5 expression as observed in peripheral blood mononuclear cells in individuals [30], suggesting that the genotype could regulate CCR5 gene expression. The infiltration of monocytes and macrophages is reportedly increased as the glomerulosclerosis progresses [31, 32]. As CCR5 expression increases, the recruitment of monocytes and differentiation of these cells to macrophage in glomeruli will also be induced in manifolds. In the present study, none of the statistic models shows any association (p>0.05) among the Malays and Indians. The result is in agreement with the Caucasians studies in Irish T1DM [33] and T2DM in Danish, Finnish, and French [34]. The allelic frequency is significantly different between different ethnic groups. An SNP that is essentially benign in one ethnic group can be very problematic in another. This can be due, for example, to differences in the frequencies of other SNPs as well as epigenetic differences such as methylation due to diet.

The proinflammatory chemokine IL8 is important in the regulation of the inflammatory response. The A allele in the regulatory region upregulates the gene expression and increases IL8 levels [20]. IL8 is detectable in the urine when the plasma level is high [35]. It has been earlier reported that rs4073 is associated with the development of DN among T2DM in the North and South Indian populations [13]. In the present study, among the Malaysians, only the Indians showed a significant association of rs4073 but not the Malays and Chinese. This may be due to the same ancestral origin of the Indians. The association was significantly observed in the additive (p=0.0053) and recessive (p=0.0092) models when tested with the Cochran-Armitage trend test. The Indians have the least frequency of the protective T allele among the ethnic groups. The A allele has the ability to increase the risk of T2DM nephropathy with weak association among the Malaysian Indians.

### 4.2. CCL2 rs3917887 D>I

CCL2 is upregulated and directly involved in the pathogenesis of DN [36]. This research examined the insertion/deletion of the CCL2 gene for association with DN but showed no significant association among Malaysians. The result disagrees with the finding of Ahluwalia et al. [13] among the North and South Indians. Though there was no significant association of rs3917887 with T2DM nephropathy among the Malays, Chinese, and Indians, the Indians had the highest percentage of deleted microsatellites carriers in the case subjects (63.1%) compared to the control (40.0%). The differences of deleted microsatellites carriers' frequency among the Malays in case (55.0%) and control (42.2%) were similar to the Chinese's case (49.5%) and control (45.3%).

### 4.3. ELMO1 rs741301 A>G

SNP rs741301 increases the production of ELMO1 which promotes phagocytosis, with excessive production of extracellular protein (type 1 collagen and fibronectin), and diminishes cell adherence [24, 28, 37], thereby causing the development and progression of T2DM glomerulosclerosis. The control sample of Shimazaki et al. [28] was not in HWE as noted by Sulgi et al. [38], due to systematic differential bias in genotyping or population stratification within their samples. The same association was also found among the Chinese [26] and African Americans [27]. In the present study, there was no significant statistical association of rs741301 with T2DM nephropathy when the genotype was initially tested by conventional Chi^2^ test with df=2. Both control and case are in the HWE (p>0.05). Therefore, a null hypothesis was statistically accepted when tested with the other three models. Similar observation of no association was reported by [39]. Therefore SNP rs741301 is not a contributing risk factor to T2DM nephropathy among the Malaysian population.

## 5. Conclusion

An inflammatory process involving immune cells plays significant roles in the development and pathogenesis of DN among T2DM. Among the four genetic polymorphisms evaluated in the present study (CCL2-rs3917887, CCR5-rs1799987, ELMO1-rs74130, and IL8-rs4073), rs4073 (T>A) of IL8 is associated with the Indians only while rs1799987 (G>A) of CCR5 was associated with Chinese. The additive model was the best model for the mode of inheritance of all the genes. Nevertheless, the contribution of genetic variants differs across ethnic groups or background.

## Figures and Tables

**Table 1:** The sequence of primers and size of the PCR products used for the genotyping.

No	SNP	Forward	Reverse	PCR Products (bp)	Tm (°C)
1	rs1799987	ACGTTGGATGATACGGGGAGAGTGGAGAAA	ACGTTGGATGCCAACTTTAAATGTAGAGGG	95	47.0
2	rs3917887	ACGTTGGATGCCTATGCTGTAAAATGGGTA	ACGTTGGATGGTCCGTCTTAATGACACTTG	117	45.4
3	rs4073	ACGTTGGATGCTGAAGCTCCACAATTTGGT	ACGTTGGATGGCCACTCTAGTACTATATCTG	118	45.2
4	rs741301	ACGTTGGATGCAGTTCCCATGGTGGTTATC	ACGTTGGATGGAACTCTTCAAGCTCAATAG	110	46.1

**Table 2:** Comparison of clinical characteristic across the races.

SNP	Nephropathy				Without Nephropathy			
	Malay	Chinese	Indian	p-value	Malay	Chinese	Indian	p-value
				df=2				df=2
Albumin excretion rate (g/24hr)	1363.18±136.00	1952.50±144.30	1756±155.44	0.77	25.83±2.10	23.00±5.70	26.67±1.9	0.61
Glycated hemoglobin (%)	8.67±2.34	9.19±2.31	7.79±2.08	<0.01^*∗*^	9.07±1.97	9.45±2.56	8.30±1.63	<0.01^*∗*^
Fasting blood glucose (mmol/L)	9.91±3.3	9.60±2.10	9.8±0.137	0.17	9.86±3.70	8.6±3.70	8.78±3.69	0.61
Total cholesterol (mmol/L)	6.42±1.37	6.58±1.19	6.48±1.42	0.11	4.84±1.01	4.26±1.13	4.74±1.23	0.021
HDL cholesterol (mmol/L)	1.02±0.39	1.01±0.24	1.16±0.25	0.13	1.22±0.26	1.11±0.24	1.29±0.26	0.14
LDL cholesterol (mmol/L)	2. 45±1.25	2.69±1.05	2.61±1.25	0.22	2.82 ±1.10	2.53±0.90	2.60±1.06	0.11
Triglycerides (mmol/L)	1.85±0.80	1.72±0.64	1.55±0.67	0.12	2.07±1.81	1.36±0.80	1.87±0.87	0.16

^*∗*^p<0.05 shows significant difference.

**Table 3:** Genotype distribution and frequencies in case and control.

SNP	Malay						Chinese						Indian					
	Case			Control			Case			Control			Case			Control		
	major/ major	major/ minor	minor/ minim	major/ major	major/ minor	minor/ minor	major/ major	major/ minor	minor/ minor	major/ major	major/ minor	minor/ minor	major/ major	major/ minor	minor/ minor	major/ major	major/ minor	minor/ minor
CCR5 rs1799987	GG=47 (35.9)	AG=69 (52.7)	AA=15 (11.5)	GG=43 (44.8)	AG=42 (43.8)	AA=11 (11.5)	GG=25 (23.1)	AG=58 (53.7)	AA=25 (23.1)	GG=47 (49.5)	AG=41 (43.2)	AA=7 (7.4)	GG=29 (33.7)	AG=44 (51.2)	AA=13 (15.1)	GG=48 (35.3)	AG=70 (51.5)	AA=18 (13.2)
CCL2 rs3917887	II=42 (32.1)	DI=57 (43.5)	DD=32 (24.4)	II=32 (33.3)	DI=47 (44.0)	DD=17 (20.7)	II=20 (18.5)	DI=69 (63.9)	DD=19 (17.6)	II=26 (27.4)	DI=57 (54.7)	DD=17 (17.9)	II=33 (38.8)	DI=40 (47.1)	DD=12 (14.1)	II=41 (31.2)	DI=62 (51.7)	DD=17 (14.1)
IL8 rs4073	TT=45 (34.4)	TA=66 (50.4)	AA=20 (15.3)	TT=45 (34.4)	TA=66 (50.4)	AA=14 (15.3)	TT=43 (39.8)	TA=57 (52.8)	AA=8 (7.4)	TT=35 (36.8)	TA=50 (52.6)	AA=10 (10.5)	TT=23 (26.7)	TA=51 (59.3)	AA=12 (14.0)	TT=60 (44.1)	TA=56 (41.2)	AA=20 (14.7)
ELMO1 rs741301	AA=50 (38.2)	AG=58 (44.3)	GG=23 (17.6)	AA=82 (36.1)	AG=108 (47.6)	GG=37 (16.3)	AA=40 (37.0)	AG=51 (47.2)	GG=17 (15.7)	AA=42 (44.2)	AG=44 (46.3)	GG=9 (9.5)	AA=33 (38.4)	AG=45 (52.3)	GG=8 (9.3)	AA=57 (41.9)	AG=59 (43.4)	GG=20 (14.7)

Genotype data are presented as a number of subjects (%).

**Table 4:** Allele distribution and frequencies in case and control.

SNP	Malay				Chinese				Indian			
	Case		Control		Case		Control		Case		Control	
CCR5 rs1799987	G=163 (62.2)	A=99 (37.8)	G=128 (66.7)	A=64 (33.3)	G=108 (50)	A=108 (50)	G=102 (71.1)	A=55 (28.9)	G=102 (59.3)	A=70 (40.7)	G=166 (61)	A=106 (39.0)
CCL2 rs3917887	I=99 (45.0)	D=121 (55.0)	I=111 (57.8)	D=81 (42.2)	I=109 (50.5)	D=107 (49.5)	I=104 (54.7)	D=86 (45.3)	I=62 (36.9)	D=106 (63.1)	I=144 (60.0)	D=96 (40.0)
IL8 rs4073	T=156 (59.4)	A=106 (40.6)	T=122 (63.5)	A=70 (36.5)	T=156 (68.0)	A=73 (32.0)	T=120 (63.2)	A=70 (36.8)	T=97 (56.4)	A=75 (43.6)	T=176 (64.7)	A=96 (35.3)
ELMO1 rs741301	A=158 (60.3)	G=104 (36.7)	A=114 (59.4)	G=78 (40.6)	A=131 (60.6)	G=85 (39.4)	A=128 (67.4)	G=62 (32.6)	A=111 (64.5)	G=61 (35.5)	A=173 (63.6)	G=99 (36.4)

Allele data are presented as a number of subjects (%).

**Table 5:** Differences in the frequencies of allele distribution among the races.

SNP	Control							Case						
	Malay		Chinese		Indian		P value	Case		Control		Indian		P value
							*χ*2							*χ*2
							df=2							
														df=2
CCR5	G=128	A=64	G=102	A=55	G=166	A=106	0.432	G=163	A=99	G=108	A=108	G=102	A=70	**0.023** ^**∗**^
rs1799987	(66.7)	(33.3)	(71.1)	(28.9)	(61)	(39.0)	1.68	(62.2)	(37.8)	(50)	(50)	(59.3)	(40.7)	7.57
CCL2	I=111	D=81	I=104	D=86	I=144	D=96	1.200	I=99	D=121	I=109	D=107	I=62	D=62	**<0.001** ^**∗**^
rs3917887	(57.8)	(42.2)	(54.7)	(45.3)	(60.0)	(40.0)	0.549	(45.0)	(55.0)	(50.5)	(49.5)	(36.9)	(63.1)	28.24
IL8	T=122	A=70	T=120	A=70	T=176	A=96	0.9371	T=156	A=106	T=156	A=73	T=97	A=75	**0.004** ^**∗**^
rs4073	(63.5)	(36.5)	(63.2)	(36.8)	(64.7)	(35.3)	0.13	(59.4)	(40.6)	(68.0)	(32.0)	(56.4)	(43.6)	6.56
ELMO1	A=114	G=78	A=128	G=62	A=173	G=99	0.2671	A=158	G=104	A=131	G=85	A=111	G=61	0.641
rs741301	(59.4)	(40.6)	(67.4)	(32.6)	(63.6)	(36.4)	2.34	(60.3)	(36.7)	(60.6)	(39.4)	(64.5)	(35.5)	0.89

^*∗*^p<0.05 indicates the significant difference of allele distribution in the population.

**Table 6:** Hardy Weinberg Equilibrium test for the control and case.

SNP	Malay						Chinese						Indian					
	Control			Statistic			Control			Statistic			Control			Statistic		
	major/ major	major/ minor	minor/ minor	*χ*2	p-value	df	major/ major	major/ minor	minor/ minor	*χ*2	p-value	df	major/ major	major/ minor	minor/ minor	*χ*2	p-value	df
**CONTROL**																		

CCR5 rs1799987	GG=43 (44.8)	AG=42 (43.8)	AA=11 (11.5)	0.023	0.8795	1	GG=47 (49.5)	AG=41 (43.2)	AA=7 (7.4)	0.229	0.6313	1	GG=48 (35.3)	AG=70 (51.5)	AA=18 (13.2)	0.916	0.3385	1
CCL2 rs3917887	II=32 (33.3)	DI=47 (44.0)	DD=17 (20.7)	0.001	0.9745	1	II=26 (27.4)	DI=57 (54.7)	DD=17 (17.9)	2.229	0.1354	1	II=41 (31.2)	DI=62 (51.7)	DD=17 (14.1)	0.700	0.4028	1
IL8 rs4073	TT=45 (34.4)	TA=66 (50.4)	AA=14 (15.3)	1.960	0.1615	1	TT=35 (36.8)	TA=50 (52.6)	AA=10 (10.5)	1.629	0.2018	1	TT=60 (44.1)	TA=56 (41.2)	AA=20 (14.7)	1.319	0.2508	1
ELMO1 rs741301	AA=82 (36.1)	AG=10 8 (47.6)	GG=37 (16.3)	0.021	0.8848	1	AA=42 (44.2)	AG=44 (46.3)	GG=9 (9.5)	0.271	0.6027	1	AA=57 (41.9)	AG=59 (43.4)	GG=20 (14.7)	0.540	0.4624	1

**CASE**																		

CCR5 rs1799987	GG=47 (35.9)	AG=69 (52.7)	AA=15 (11.5)	1.895	0.1686	1	GG=25 (23.1)	AG=58 (53.7)	AA=25 (23.1)	0.5926	0.4414	1	GG=29 (33.7)	AG=44 (51.2)	AA=13 (15.1)	0.309	0.0788	1
CCL2 rs3917887	II=42 (32.1)	DI=57 (43.5)	DD=32 (24.4)	2.036	0.1536	1	II=20 (18.5)	DI=69 (63.9)	DD=19 (17.6)	8.340	0.0039^*∗*^	1	II=33 (38.8)	DI=40 (47.1)	DD=12 (14.1)	0.001	0.9748	1
IL8 rs4073	TT=45 (34.4)	TA=66 (50.4)	AA=20 (15.3)	0.273	0.0601	1	TT=43 (39.8)	TA=57 (52.8)	AA=8 (7.4)	3.477	0.0622	1	TT=23 (26.7)	TA=51 (59.3)	AA=12 (14.0)	3.641	0.0564	1
ELMO1 rs741301	AA=50 (38.2)	AG=58 (44.3)	GG=23 (17.6)	0.741	0.3893	1	AA=40 (37.0)	AG=51 (47.2)	GG=17 (15.7)	0.012	0.9117	1	AA=33 (38.4)	AG=45 (52.3)	GG=8 (9.3)	1.761	0.1845	1

p>0.05 shows consistency with HWE. Genotype data are presented as a number of subjects (%).

**Table 7:** Association of polymorphism in T2DM with and without nephropathy.

		**Malay**				**Chinese**				**Indian**			
		Multiplicative model		Dominant Model	Recessive Model	Multiplicative model		Dominant Model	Recessive Model	Multiplicative model		Dominant Model	Recessive Model
		Genotype (df=2)	Allele (df=1)	Major/ Major vs. others (df=1)	Minor/ Minor vs. others (df=1)	Genotype (df=2)	Allele (df=1)	Major/ Major vs. others (df=1)	Minor/ Minor vs. others (df=1)	Genotype (df=2)	Allele (df=1)	Major/ Major vs. others (df=1)	Minor/ Minor vs. others (df=1)
CCR5 rs1799987	*χ* ^2^	2.012 0.3656	0.955 0.3284	1.840 0.1749	0.0001 0.9920	19.012 **7.4x**10^−5**∗**^	18.645 **1.6x**10^−5**∗**^	15.304 **9.2x**10^−5**∗**^	9.478 **0.0021**^**∗**^	0.172 0.9176	0.131 0.7174	0.058 0.8096	0.155 0.6938
	p												
CCL2 rs3917887	*χ*2	1.545 0.4618	6.735 0.0094	0.041 0.8395	1.478 0.2241	2.460 0.2923	0.740 0.3897	2.259 0.1328	0.003 0.9563	0.511 0.7745	0.399 0.5276	0.468 0.4939	0.0001 0.9920
	p												
IL8	*χ*2	1.321	0747	1.266	0.200	0.671	1.1384	0.189	0.608	7.865	3.073	0.024	0.173
rs4073	p	0.5165	0.3874	0.2605	0.6547	0.7150	0.2859	0.6638	0.4355	0.0196^**∗**^	0.0796	0.8769	0.6775
ELMO1 rs741301	*χ* ^2^	1.369 0.5043	0.040 0.8414	0.561 0.4538	0.359 0.5491	2.203 0.332	1.976 0.1598	0.011 0.9165	1.080 0.2986	2.282 0.3195	0.040 0.8414	0.274 0.6007	1.396 0.2374
	p												

^*∗*^p<0.05 indicates an association of polymorphisms and disease in a different mode of inheritance.

**Table 8:** Cochran-Armitage trend testing.

		**Malay**				**Chinese**				**Indian**			
		Multiplicative df=1	Additive df=1	Dominant df=1	Recessive df=1	Multiplicative df=1	Additive df=1	Dominant df=1	Recessive df=1	Multiplicative df=1	Additive df=1	Dominant df=1	Recessive df=1
CCR5 rs1799987	*χ* ^2^	-	-	-	-	19.012 7.4x10^−5*∗*^	53.983 **1.0x**10^−15**∗**^	9.478 0.0021^*∗*^	15.303 0.00009^*∗*^	-	-	-	-
	p												
IL8 rs4073	*χ* ^2^	-	-	-	-	-	-	-	-	3.073 0.0796^*∗*^	7.769 **0.0053**^*∗*^	0.024 0.8768	6.793 0.0092^*∗*^
	p												

The mode of inheritance is best presented with the least **p-value**^*∗*^.

**Table 9:** The odd ratio of polymorphism in association with T2DM and nephropathy.

	**Malay**				**Chinese**				**Indian**			
	Allele		Genotype		Allele		Genotype		Allele		Genotype	
	Multiplicative Model a vs. A	Additive Model AA vs aa Aa vs aa	Dominant Model AA vs. Aa + aa	Recessive Model AA + Aa vs. aa	Multiplicative Model a vs. A	Additive Model AA vs aa Aa vs aa	Dominant Model AA vs. Aa + aa	Recessive Model AA + Aa vs. aa	Multiplicative Model a vs. A	Additive Model AA vs aa Aa vs aa	Dominant Model AA vs. Aa + aa	Recessive Model AA + Aa vs. aa
CCR5					2.46	6.71	0.31	3.79	-	-	-	-
rs1799987	-	-	-	-	(1.62-3.71)	(2.55-17.68)	(0.12-0.56)	(1.56-9.22)				
(G/A)						2.52						
						(0.99-6.39)						
IL8					-	-	-	-	1.42	1.57	0.46	0.94
rs4073	-	-	-	-					(0.96-2.10)	(0.66-3.71)	(0.26-0.83)	(0.43-2.04)
(T/A)										0.66		
										(0.29-1.48)		

ODD Ratio (OR), 95% CI. A: the major allele; a: the minor allele or increased risk.

## Data Availability

The data used to support the findings of this study are included in the article.
